# Relationships Between Level and Change in Sarcopenia and Other Body Composition Components and Adverse Health Outcomes: Findings from the Health, Aging, and Body Composition Study

**DOI:** 10.1007/s00223-020-00775-3

**Published:** 2020-11-15

**Authors:** Leo D. Westbury, Holly E. Syddall, Nicholas R. Fuggle, Elaine M. Dennison, Nicholas C. Harvey, Jane A. Cauley, Eric J. Shiroma, Roger A. Fielding, Anne B. Newman, Cyrus Cooper

**Affiliations:** 1 MRC Lifecourse Epidemiology Unit, University of Southampton, Southampton, UK; 2 Victoria University of Wellington, Wellington, New Zealand; 3 NIHR Southampton Biomedical Research Centre, University of Southampton and University Hospital Southampton NHS Foundation Trust, Southampton, UK; 4 Department of Epidemiology, Graduate School of Public Health, University of Pittsburgh, Pittsburgh, USA; 5 Laboratory of Epidemiology and Population Sciences, Intramural Research Program, National Institute on Aging, Baltimore, USA; 6 Nutrition, Exercise Physiology, and Sarcopenia Laboratory, Jean Mayer USDA Human Nutrition Research Center on Aging, Tufts University, Boston, USA; 7 NIHR Oxford Biomedical Research Centre, University of Oxford, Oxford, UK

**Keywords:** Epidemiology, Sarcopenia, Osteoporosis, Aging, Older people

## Abstract

We investigated how baseline values and rates of decline in components of sarcopenia and other body composition parameters relate to adverse clinical outcomes using the Health, Aging, and Body Composition Study. 2689 participants aged 70–79 years were studied. Appendicular lean mass, whole body fat mass, and total hip BMD were ascertained using DXA; muscle strength by grip dynamometry; and muscle function by gait speed. Baseline values and 2–3 year conditional changes (independent of baseline) in each characteristic were examined as predictors of mortality, hospital admission, low trauma fracture, and recurrent falls in the subsequent 10–14 years using Cox regression (generalized estimating equations used for recurrent falls) with adjustment for sex, ethnicity, age, and potential confounders. Lower levels and greater declines in all parameters (excluding hip BMD level) were associated (*p* < 0.05) with increased rates of mortality; fully-adjusted hazard ratios per SD lower gait speed and grip strength were 1.27 (95% CI 1.19, 1.36) and 1.14 (1.07, 1.21), respectively. Risk factors of hospital admission included lower levels and greater declines in gait speed and grip strength, and greater declines in hip BMD. Lower levels and greater declines in fat mass and hip BMD were associated with low trauma fracture. Lower gait speed, higher fat mass, and both lower levels and greater declines in grip strength were related to recurrent falls. Lower baseline levels and greater declines in musculoskeletal parameters were related to adverse outcomes. Interventions to maximize peak levels in earlier life and reduce rates of age-related decline may reduce the burden of disease in this age group.

**Electronic supplementary material:**

The online version of this article (10.1007/s00223-020-00775-3) contains supplementary material, which is available to authorized users.

## Introduction

Sarcopenia, the accelerated loss of skeletal muscle mass and strength with age, is associated with physical disability, mortality, and significant healthcare expenditure [1]. Although there is no consensus algorithm for diagnosing sarcopenia, the recent convergence in definitions of sarcopenia recognizes muscle mass (appendicular lean mass), strength (grip strength), and function (gait speed) as key components [2, 3].

Previous research has examined levels and changes in some sarcopenia components in relation to adverse health outcomes. For example, the relationship between lower levels and greater declines in gait speed and grip strength in relation to greater risk of mortality has been reported previously [4–6]. However, there is uncertainty with regard to the strength of associations between baseline values and subsequent rates of decline in these measures in relation to clinical outcomes in older people. Furthermore, to our knowledge, no studies have explored both baseline values and changes in sarcopenia components in relation to multiple adverse health outcomes among a single cohort of community-dwelling older people.

Many cohort studies have suggested four critical adverse health outcomes associated with sarcopenia in older people: death (Third National Health and Nutrition Examination Survey [7] and InCHIANTI Study [8]), hospitalization (InCHIANTI Study [8]), fracture (Osteoporotic Fractures in Men (MrOS) Study [9]), and falls (Healthy Ageing Initiative Cohort Study [10]). Accordingly, in this paper, we examine baseline values and changes in sarcopenia components in relation to these outcomes among participants in the Health, Aging, and Body Composition (Health ABC) Study, USA. For completeness, levels and changes in other aspects of body composition, namely fat mass and bone mineral density, are also included as predictors.

## Methods

### The Health, Aging, and Body Composition Study

The Health ABC Study comprises 3075 US men and women (aged 70–79 years at baseline) who were recruited in 1997–1998 [11]. A random sample of white and all the black Medicare beneficiaries from around Memphis (Tennessee) and Pittsburgh (Pennsylvania) was obtained. Sampled participants received a mailing followed by a telephone eligibility screen. The original purpose of the Health ABC Study was to understand risk factors for the decline of function and change in body composition among healthy older people [12]. Therefore, the cohort was selected to be free of mobility limitation at baseline. Individuals reporting no difficulty in walking one quarter of a mile or climbing 10 stairs were considered eligible. Individuals with the following characteristics were excluded: inability to communicate with the interviewer; clear cognitive impairment; having a life-threatening illness or difficulties with activities of daily living (ADL); requiring a walking aid; having an intention of moving outside the area within three years; or currently enrolled in a lifestyle intervention trial. Written informed consent was provided by all participants and the study was approved by the institutional review boards at the University of Tennessee and the University of Pittsburgh. Initially, participant information was ascertained from annual examinations from baseline (Year 1: 1997–1998) to Year 6 (2002–2003) and from biannual questionnaires from baseline to Year 14 (2010–2011).

### Ascertainment of Sarcopenia and Other Body Composition Parameters

Gait speed was ascertained at Years 2 (1998–1999) and 4 (2000–2001) by asking participants to walk at their normal speed down a corridor over a total distance of 20 m. Grip strength was measured twice for each hand at Years 1 and 4 using a Jamar hydraulic dynamometer with the participant in the sitting position with the arm to be tested resting on the table and the elbow held at approximately a right angle, according to a standardized protocol [13]; the calibration of the dynamometers was checked regularly. Maximum grip strength at each time point was used for analyses. Grip strength values were set to missing for participants with severe hand pain or recent surgery. Whole body dual-energy X-ray absorptiometry scans (Hologic QDR 4500A; Hologic, Bedford, MA, USA) were performed at Years 1 and 4 and used to ascertain whole body fat and appendicular lean mass (ALM). Total hip BMD was measured using the same device at Years 1 and 3 (1999–2000). The reproducibility and validity of this scanner have been previously reported [14, 15]. Regular DXA phantom scans were performed for quality control and calibration purposes.

### Ascertainment and Derivation of Potential Confounders

The study methodology has been described in detail previously [12]. In brief, at baseline (Year 1), sex, ethnicity, educational attainment, home ownership, and health behaviors such as smoking status and alcohol consumption were self-reported using questionnaires. Height and weight were measured using a Harpenden Stadiometer (Holtain Ltd, Crosswell, UK) and a standard balance beam scale, respectively. Height and weight were highly correlated (*r* = 0.45, *p* < 0.001 for men; *r* = 0.31, *p* < 0.001 for women); to avoid multi-collinearity in models, a sex-specific standardized residual of weight-adjusted-for-height was derived as a measure of adiposity. Physical activity over the past 7 days was assessed using an interviewer-administered questionnaire. Approximate metabolic equivalent unit values were assigned to reported activities and intensity levels to derive caloric expenditure in kcal/kg/h [16]. Total kilocalories expended per week was calculated by multiplying the participant's weight (kg) by the sum of the caloric expenditure for all activities performed, as previously described [17]. As in previous analyses [18, 19], the number of self-reported comorbidities (ever told by a doctor) was used as a marker of comorbidity out of the following: stroke, diabetes, Parkinson’s disease, chronic obstructive pulmonary disease, heart attack or myocardial infarction, congestive heart failure and hypertension. Cognitive function was measured using the Modified Mini-Mental State Examination (3MS) with scores ranging from 0 to 100 (higher scores indicate better function) [20]; scores were dichotomized for analysis; and participants with scores < 80 were regarded as having low cognitive function [21].

At Year 2, dietary intake over the previous year was assessed using a nurse-administered food frequency questionnaire (FFQ) comprising 108 items. To assess the extent to which Health ABC participants’ diets conformed to recommendations of the Dietary Guidelines for Americans of 1995 and the Food Guide Pyramid of 1992, a healthy eating index (HEI), ranging from 0 to 100, was calculated for each participant; higher scores reflected healthier diets [22].

### Ascertainment of Adverse Outcomes

Deaths from baseline until 30th September 2014 were determined from death certificates, hospital records, and interviews with next of kin. All deaths were adjudicated by a central committee. Participants reported hospital admissions during follow-up and were asked specific questions about their admissions every 6 months [23]. Medical records for each reported admission were collected; these contained information on admission and discharge dates and the main reason for admission. Information on diagnoses and length of stay were checked by local review. Fractures were ascertained by self-report every 6 months and confirmed by radiology reports. For this analysis, fracture events were limited to low trauma fractures, defined as 'spontaneous or with modest trauma, such as a fall from a standing height' [24]. Adjudication for admissions and fractures was complete until 14th August 2012; events occurring after this date were not used for this analysis. At every year of follow-up, up to and including Year 14 (2010–2011), participants reported the number of times they had fallen over and landed on the floor or the ground during the last 12 months; an indicator variable for recurrent falls (≥ 2) was derived at each year of follow-up.

### Derivation of Exposures

For gait speed, grip strength, ALM, fat mass, and hip BMD, exposures were baseline levels at Year 1 (Year 2 for gait speed) and conditional changes from Years 1 to 4 (2 to 4 for gait speed and 1 to 3 for hip BMD). Conditional changes (independent of baseline) were characterized by residuals from sex-specific linear regression models for parameters at follow-up on baseline parameters with adjustment for individual follow-up duration.

### Statistical Methods

Baseline participant characteristics were described using means and standard deviations (SD), medians and inter-quartile ranges and frequency and percentage distributions. For each parameter, outcomes only included adverse events (deaths, hospital admissions, low trauma fractures, and recurrent falls) occurring after Year 4 (Year 3 for hip BMD). Exposures were examined in relation to death, hospital admission, and low trauma fracture using time-to-first-event Cox proportional hazards models with death as a censoring event for the latter two. For those who did not experience the adverse event and did not leave the study early, time at risk ended on 30th September 2014 for death and 14th August 2012 for hospital admission and low trauma fracture. Exposures in relation to risk of recurrent falls were examined using a generalized estimating equations (GEE) model, as in previous studies [25–27], with a binomial distribution, logit link function, and robust standard errors to account for clustering within individuals.

Adjustments were selected a priori and included measures of anthropometric, sociodemographic, lifestyle, and clinical characteristics that are known to influence both the sarcopenia and other body composition components [28] and risk of the adverse health outcomes considered. All models were adjusted for baseline age and for a four-level variable reflecting the possible combinations of sex and ethnicity; there was no evidence of interactions between exposures and this four-level variable. Fully-adjusted models also accounted for height, weight-for-height residual (not used in models for level relating to ALM and fat mass due to collinearity), smoking status (ever vs never), alcohol consumption, healthy eating index, physical activity, educational attainment, home ownership, cognitive function, and number of comorbidities. Cox models for hospital admission and low trauma fracture were also stratified on whether or not participants experienced these events before the start of the survival analysis follow-up (Year 3 for hip BMD and Year 4 for the remaining parameters); similarly, GEE models for recurrent falls were adjusted for previous recurrent falls.

All analyses were based on the sample of 2689 participants with data on both baseline level and conditional change for at least one of the parameters (gait speed, grip strength, ALM, fat mass, and hip BMD). Exposures (baseline levels and conditional changes in parameters) were standardized (sex-specific) in models to enable the comparison of effect sizes. Analyses were conducted using Stata, release 15 (StataCorp, College Station, TX, USA).

Several sensitivity analyses were performed. The number of hospital admissions was analyzed using negative binomial regression with robust variance estimation as in a previous study [23]; a competing risk analysis for hospital admission and low trauma fracture was implemented, with death as a competing event, using the Fine-Gray subdistribution hazards model [29]; and cause-specific mortality (cardiovascular-related, cancer-related, and other) was examined as an outcome. Further sensitivity analyses involved examining: ALM residuals (derived from sex-specific models predicting ALM from height and fat mass as in previous analyses [30]) which reflect whether levels of ALM were higher or lower than expected, given stature and fat mass; and changes in ALM after adjusting for changes in weight as in previous analyses [31] which reflect whether declines in ALM were greater than expected, given total weight change.

## Results

### Descriptive Statistics

Baseline participant characteristics among the analysis sample of 2689 Health ABC participants according to sex and ethnicity are presented in Table 1. Mean and standard deviation (SD) for age was 74.1 (2.8) years. Women had higher fat mass, but all other components of sarcopenia and additional body composition parameters were greater among men (*p* < 0.001 for all associations). Survival analysis descriptive statistics are presented in Table 2 for the 2480 participants with data on exposures and survival analysis follow-up times starting at Year 4; these statistics differ for hip BMD where follow-up time started at Year 3. Median follow-up time (number of years from Year 4 to the first event or until participants were censored) was greater for death (11.3) and low trauma fracture (9.8) compared with hospital admission (3.4). A significantly (*p* < 0.05) higher proportion of men than women died (66.7% vs 55.3%) or had a hospital admission (87.6% vs 84.5%); a higher proportion of women than men had low trauma fractures (21.9% vs 11.0%) or recurrent falls (46.2% vs 41.6%).

**Table 1:** Baseline participant characteristics according to sex and ethnicity

Characteristic [mean (SD) or *N* (%)]	Men			Women		
	White (*n* = 846)	Black (*n* = 446)	All (*n* = 1292)	White (*n* = 782)	Black (*n* = 615)	All (*n* = 1397)
Age (years)	74.3 (2.9)	74.1 (2.8)	74.2 (2.8)	74.0 (2.8)	73.8 (2.9)	73.9 (2.9)^†^
Height (m)	1.73 (0.06)	1.73 (0.07)	1.73 (0.07)	1.60 (0.06)	1.60 (0.06)	1.60 (0.06)
Weight (kg)	81.5 (12.4)	81.7 (14.3)	81.6 (13.1)	66.2 (12.1)	75.7 (15.8)	70.4 (14.6)^†^
BMI (kg/m^2^)	27.0 (3.7)	27.2 (4.3)	27.1 (3.9)	26.0 (4.5)	29.7 (5.9)	27.6 (5.4)^†^
Ever smoked	592 (70.1%)	306 (68.6%)	898 (69.6%)	316 (40.4%)	269 (43.8%)	585 (41.9%)
Current drinker	544 (64.6%)	205 (46.3%)	749 (58.3%)^†^	415 (53.2%)	191 (31.1%)	606 (43.5%)^†^
Physical activity (Mcal/week)	5.7 (3.2, 8.9)	4.7 (2.4, 9.1)	5.4 (3.0, 8.9)	4.6 (2.8, 7.3)	4.6 (2.6, 7.6)	4.6 (2.7, 7.5)
Healthy eating index^a^	70.6 (11.5)	63.7 (12.1)	68.3 (12.1)^†^	72.6 (11.7)	68.5 (11.8)	70.8 (11.9)^†^
Post-secondary education	507 (60.0%)	120 (27.0%)	627 (48.6%)^†^	377 (48.3%)	170 (27.8%)	547 (39.3%)^†^
Home ownership (does not own)	147 (17.8%)	111 (25.1%)	258 (20.3%)^†^	205 (26.8%)	220 (36.3%)	425 (31.0%)^†^
Number of comorbidities^b^:						
0	363 (45.0%)	141 (33.5%)	504 (41.0%)^†^	382 (50.5%)	154 (26.6%)	536 (40.1%)^†^
1	305 (37.8%)	170 (40.4%)	475 (38.7%)^†^	306 (40.5%)	290 (50.0%)	596 (44.6%)^†^
2	120 (14.9%)	92 (21.9%)	212 (17.3%)^†^	55 (7.3%)	110 (19.0%)	165 (12.4%)^†^
3/4	19 (2.4%)	18 (4.3%)	37 (3.0%)^†^	13 (1.7%)	26 (4.5%)	39 (2.9%)^†^
Cognitive function (3MS score)	94.0 (90.0, 97.0)	87.0 (79.5, 92.5)	92.0 (86.0, 96.0)^†^	95.0 (92.0, 97.0)	89.0 (83.0, 94.0)	93.0 (88.0, 96.0)^†^
Gait speed (m/s)^a^	1.24 (0.19)	1.10 (0.20)	1.19 (0.20)^†^	1.16 (0.19)	1.02 (0.19)	1.10 (0.20)^†^
Grip strength (kg)	39.8 (7.7)	43.0 (8.5)	40.9 (8.1)^†^	23.7 (5.1)	26.6 (6.3)	25.0 (5.8)^†^
ALM (kg)	23.3 (3.2)	25.1 (3.9)	23.9 (3.5)^†^	15.3 (2.4)	18.3 (3.2)	16.6 (3.1)^†^
Fat mass (kg)	24.8 (6.9)	23.4 (7.3)	24.3 (7.1)^†^	27.1 (7.9)	31.6 (10.1)	29.1 (9.3)^†^
Hip BMD (g/cm^2^)	0.95 (0.14)	1.02 (0.15)	0.97 (0.15)^†^	0.77 (0.13)	0.86 (0.15)	0.81 (0.15)^†^

*SD* standard deviation, *ALM* appendicular lean mass, *BMD* bone mineral density, *3MS* modified mini-mental state examination

^†^Statistically significant ethnic differences within sex (*p* < 0.05); differences between sexes were significant (*p* < 0.05) for all characteristics

^a^Ascertained at year 2

^b^Median (lower quartile, upper quartile) number of the following comorbidities (ever told by doctor): stroke, diabetes, Parkinson’s disease, chronic obstructive pulmonary disease, heart attack or myocardial infarction, congestive heart failure, and hypertension

**Table 2:** Descriptive statistics for analysis of adverse health outcomes

Characteristic [mean (SD) or *N* (%)]	Men			Women		
	White (*n* = 789)	Black (*n* = 398)	All (*n* = 1187)	White (*n* = 732)	Black (*n* = 561)	All (*n* = 1293)
Death						
Incidence	510 (64.6%)	282 (70.9%)	792 (66.7%)*^†^	387 (52.9%)	328 (58.5%)	715 (55.3%)*^†^
Follow-up time (years)	10.6 (6.5, 13.5)	9.2 (4.5, 13.3)	10.2 (5.8, 13.4)* ^†^	12.4 (8.6, 13.6)	11.4 (7.1, 13.5)	12.1 (7.9, 13.6)* ^†^
Hospital admission						
Incidence	695 (88.1%)	345 (86.7%)	1040 (87.6%)*	617 (84.3%)	476 (84.8%)	1093 (84.5%)*
Follow-up time (years)	3.0 (1.2, 6.3)	2.9 (1.2, 5.4)	2.9 (1.2, 6.0)*	4.3 (1.8, 8.4)	3.4 (1.3, 7.5)	3.9 (1.6, 8.1)* ^†^
Prevalence before follow-up	305 (38.7%)	154 (38.7%)	459 (38.7%)*	205 (28.0%)	204 (36.4%)	409 (31.6%)*^†^
Low trauma fracture						
Incidence	106 (13.7%)	23 (5.8%)	129 (11.0%)*^†^	209 (29.8%)	63 (11.6%)	272 (21.9%)*^,†^
Follow-up time (years)	9.8 (5.7, 11.5)	8.7 (4.3, 11.4)	9.6 (5.2, 11.5)^†^	10.0 (5.0, 11.6)	10.2 (5.6, 11.6)	10.1 (5.2, 11.6)
Prevalence before follow-up	16 (2.1%)	3 (0.8%)	19 (1.6%)*	42 (6.0%)	14 (2.6%)	56 (4.5%)*^,†^
Recurrent falls						
Incidence	347 (45.6%)	124 (33.5%)	471 (41.6%)*^,†^	342 (47.8%)	239 (44.1%)	581 (46.2%)*
Prevalence before follow-up	162 (21.3%)	61 (16.5%)	223 (19.7%)	147 (20.5%)	115 (21.2%)	262 (20.8%)

Of the entire study sample, 265/3075 (8.6%) died before Year 4. 256 (63.8%) of those who had a low trauma fracture also died during follow-up; figures for hospital admission and recurrent falls were 1369 (64.2%) and 662 (62.9%). Statistics presented for follow-up time starting at Year 4 among 2480 individuals with level and change measures for at least one of the following parameters: gait speed, grip strength, ALM, and fat mass; these statistics differ for hip BMD as the exposure where follow-up time started at Year 3. Individuals with no recurrent falls and missing responses for recurrent falls at Year 4 and after (*n* = 713) were regarded as not having had recurrent falls for these descriptive statistics as these individuals were included in generalized estimating equations models for recurrent falls (their non-missing responses contribute information)

*Statistically significant sex differences (*p* < 0.05)

^†^Statistically significant ethnic differences within sex (*p* < 0.05)

Compared to the 386 participants who were not included in the analytical sample, both men and women in the analytical sample had fewer comorbidities at baseline and were more likely to be white, have post-secondary education and to owner occupy their home (*p* < 0.05 for all associations).

### Baseline Values and Change in Sarcopenia and Other Body Composition Parameters and Risk of Adverse Outcomes

**Fig. 1:**
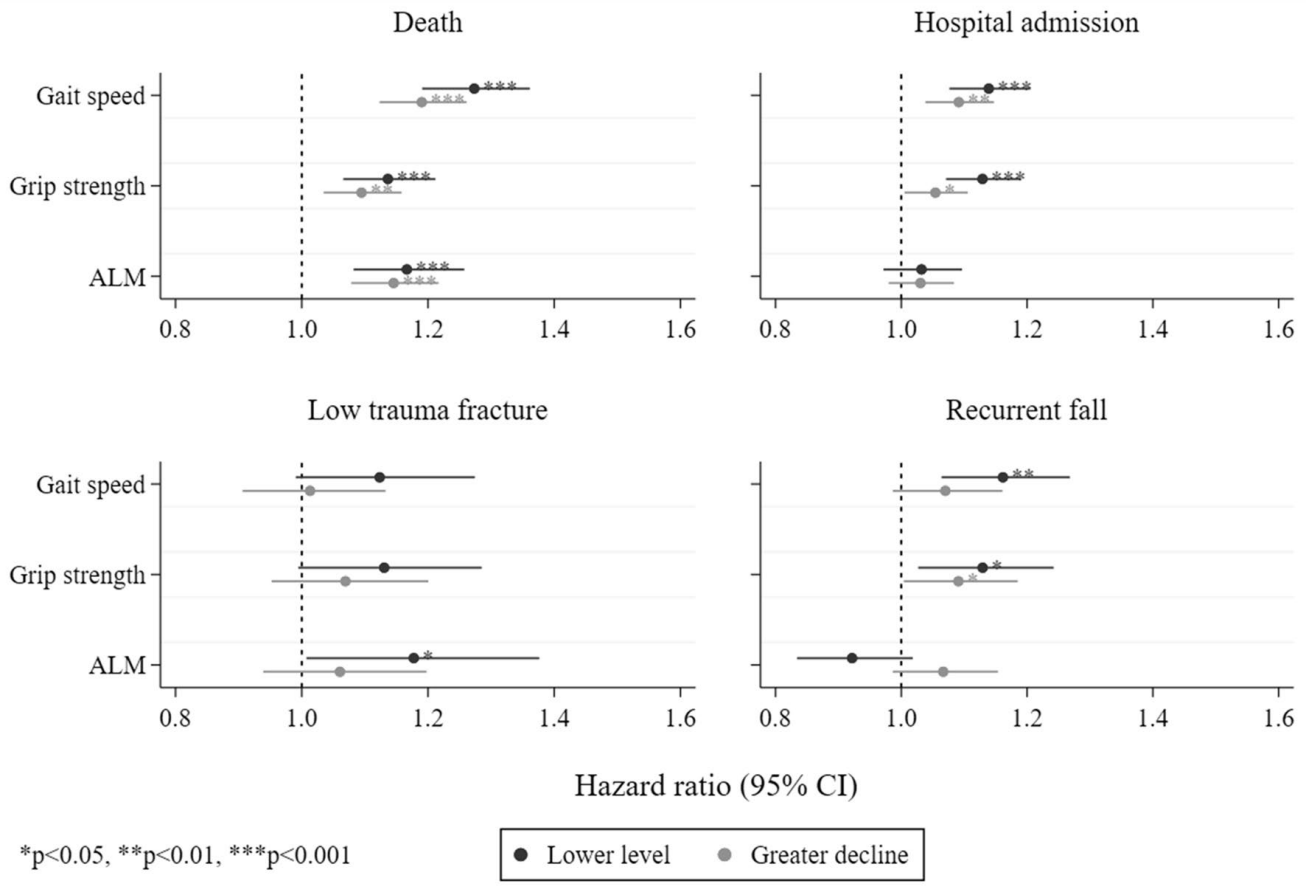
Risk of adverse outcomes per SD lower baseline level and per SD greater decline in sarcopenia component. *HR* hazard ratio (odds ratios from a generalized estimating equations model are presented for recurrent falls), *SD* standard deviation, *ALM* appendicular lean mass. Baseline levels ascertained at Year 2 for gait speed and at Year 1 for remaining predictors. Conditional changes (independent of baseline) were derived from Years 1 to 4 (Years 2 to 4 for gait speed). An indicator variable for the corresponding outcomes occurring before the survival analysis follow-up was used as the stratification variable in Cox models for low trauma fracture and hospital admission; models for recurrent falls were adjusted for previous recurrent falls. Models were adjusted for the four-level sex-ethnicity variable, age, height, weight-for-height residual (not used in models for level relating to ALM), smoking status (ever vs never), alcohol consumption, healthy eating index, physical activity, educational attainment, home ownership, cognitive function, and number of comorbidities

**Fig. 2:**
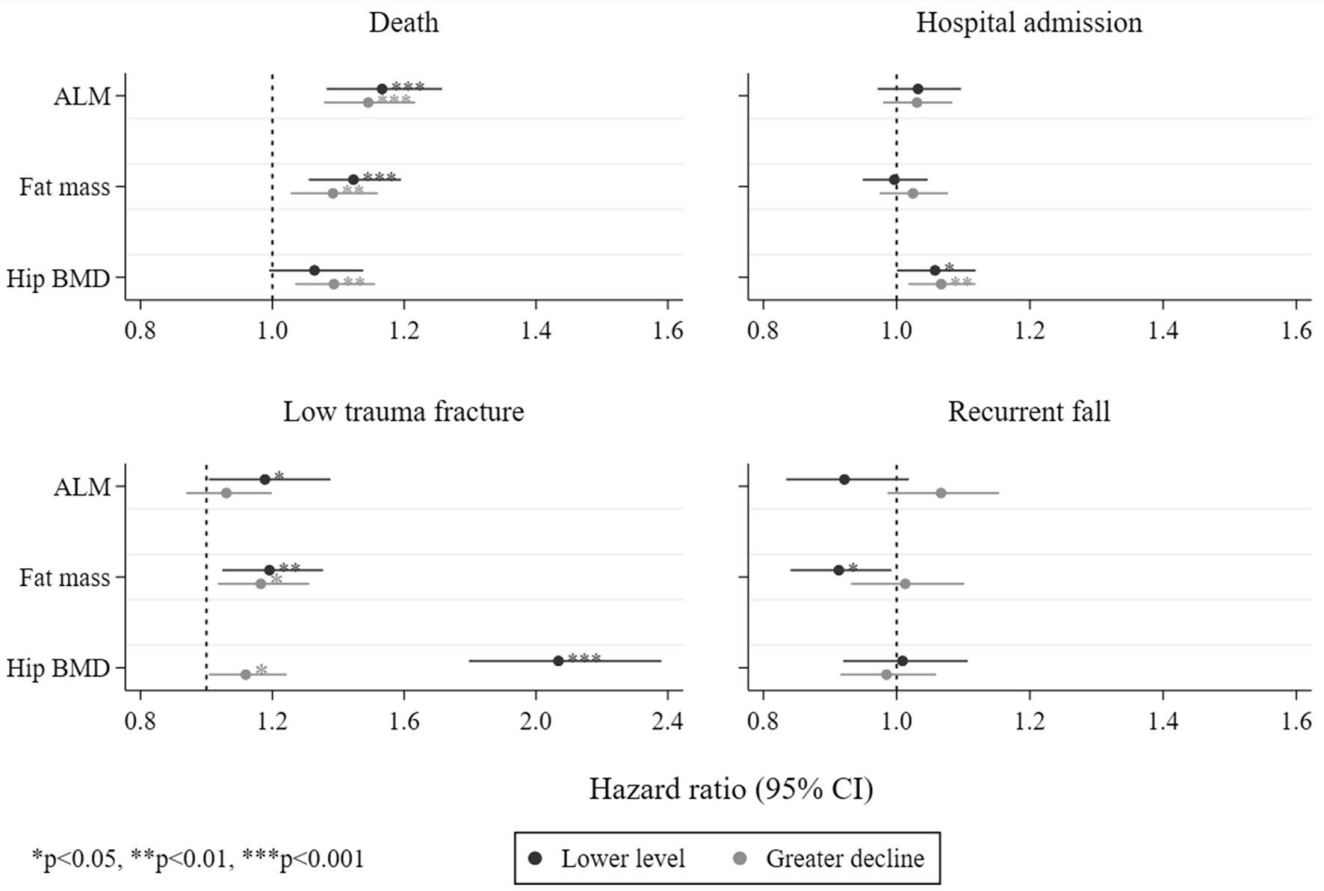
Risk of adverse outcomes per SD lower baseline level and per SD greater decline in body composition component. *HR* hazard ratio (odds ratios from a generalized estimating equations model are presented for recurrent falls), *SD* standard deviation, *ALM* appendicular lean mass, *BMD* bone mineral density. Baseline levels ascertained at Year 1. Conditional changes (independent of baseline) were derived from Years 1 to 4 (Years 1 to 3 for hip BMD). An indicator variable for the corresponding outcomes occurring before the survival analysis follow-up was used as the stratification variable in Cox models for low trauma fracture and hospital admission; models for recurrent falls were adjusted for previous recurrent falls. Models were adjusted for the four-level sex-ethnicity variable, age, height, weight-for-height residual (not used in models for level relating to ALM and fat mass), smoking status (ever vs never), alcohol consumption, healthy eating index, physical activity, educational attainment, home ownership, cognitive function, and number of comorbidities

The risk of each adverse outcome per SD lower baseline value and per SD greater decline in each parameter is presented in Tables 3 and 4, respectively. Figure 1 illustrates these associations for components of sarcopenia (gait speed, grip strength and ALM), and Fig. 2 illustrates these associations for body composition parameters (ALM, fat mass and hip BMD).

**Table 3:** Muscle function, body composition, and adverse outcomes: impact of baseline indices

Predictor	Model	Death		Hospital admission		Low trauma fracture		Recurrent fall	
		HR (95% CI)	*p*-value	HR (95% CI)	*p*-value	HR (95% CI)	*p*-value	OR (95% CI)	*p*-value
Gait speed	1	**1.31 (1.24, 1.39)**	** < 0.001**	**1.18 (1.12, 1.24)**	** < 0.001**	1.12 (1.00, 1.25)	0.059	**1.18 (1.09, 1.27)**	** < 0.001**
	2	**1.27 (1.19, 1.36)**	** < 0.001**	**1.14 (1.08, 1.21)**	** < 0.001**	1.12 (0.99, 1.27)	0.070	**1.16 (1.06, 1.27)**	**0.001**
Grip strength	1	**1.13 (1.07, 1.20)**	** < 0.001**	**1.09 (1.04, 1.14)**	** < 0.001**	**1.18 (1.05, 1.31)**	**0.004**	**1.14 (1.04, 1.24)**	**0.003**
	2	**1.14 (1.07, 1.21)**	** < 0.001**	**1.13 (1.07, 1.19)**	** < 0.001**	1.13 (0.99, 1.29)	0.061	**1.13 (1.03, 1.24)**	**0.012**
ALM	1	**1.07 (1.01, 1.14)**	**0.018**	0.99 (0.95, 1.04)	0.807	1.11 (0.99, 1.26)	0.084	0.94 (0.87, 1.02)	0.117
	2	**1.17 (1.08, 1.26)**	** < 0.001**	1.03 (0.97, 1.10)	0.306	**1.18 (1.01, 1.38)**	**0.040**	0.92 (0.83, 1.02)	0.109
Fat mass	1	**1.06 (1.00, 1.12)**	**0.036**	0.98 (0.93, 1.02)	0.291	**1.17 (1.04, 1.31)**	**0.007**	**0.91 (0.84, 0.98)**	**0.014**
	2	**1.12 (1.06, 1.20)**	** < 0.001**	1.00 (0.95, 1.05)	0.891	**1.19 (1.05, 1.35)**	**0.007**	**0.91 (0.84, 0.99)**	**0.032**
Hip BMD	1	**1.09 (1.03, 1.15)**	**0.002**	1.02 (0.98, 1.07)	0.331	**1.80 (1.61, 2.01)**	** < 0.001**	0.96 (0.89, 1.04)	0.295
	2	1.06 (0.99, 1.14)	0.070	**1.06 (1.00, 1.12)**	**0.049**	**2.07 (1.80, 2.38)**	** < 0.001**	1.01 (0.92, 1.11)	0.850

Table shows risk of adverse outcome per SD lower baseline level of predictor

Baseline levels ascertained at Year 2 for gait speed and at Year 1 for remaining predictors. An indicator variable for the corresponding outcomes occurring before the survival analysis follow-up was used as the stratification variable in Cox models for low trauma fracture and hospital admission; models for recurrent falls were adjusted for previous recurrent falls. Model 1: Adjusted for the four-level sex-ethnicity variable and age. Model 2: Additionally adjusted for height, weight-for-height residual (not used in models for ALM and fat mass), smoking status (ever vs never), alcohol consumption, healthy eating index, physical activity, educational attainment, home ownership, cognitive function, and number of comorbidities

*HR* hazard ratio (odds ratios from a generalized estimating equations model are presented for recurrent falls), *SD* standard deviation, *ALM* appendicular lean mass, *BMD* bone mineral density

Significant associations (*p* < 0.05) are highlighted in bold

**Table 4:** Muscle function, body composition, and adverse outcomes: impact of rates of loss

Predictor	Model	Death		Hospital admission		Low trauma fracture		Recurrent fall	
		HR (95% CI)	*p*-value	HR (95% CI)	*p*-value	HR (95% CI)	*p*-value	OR (95% CI)	*p*-value
Gait speed	1	**1.21 (1.14, 1.28)**	** < 0.001**	**1.11 (1.06, 1.16)**	** < 0.001**	1.03 (0.93, 1.14)	0.601	**1.08 (1.00, 1.17)**	**0.046**
	2	**1.19 (1.12, 1.26)**	** < 0.001**	**1.09 (1.04, 1.15)**	**0.001**	1.01 (0.91, 1.13)	0.818	1.07 (0.99, 1.16)	0.103
Grip strength	1	**1.14 (1.08, 1.20)**	** < 0.001**	**1.07 (1.02, 1.12)**	**0.004**	1.09 (0.98, 1.21)	0.109	**1.10 (1.02, 1.19)**	**0.016**
	2	**1.09 (1.03, 1.16)**	**0.002**	**1.05 (1.01, 1.11)**	**0.030**	1.07 (0.95, 1.20)	0.257	**1.09 (1.00, 1.19)**	**0.040**
ALM	1	**1.17 (1.11, 1.24)**	** < 0.001**	**1.05 (1.01, 1.10)**	**0.024**	1.06 (0.95, 1.19)	0.279	**1.10 (1.03, 1.19)**	**0.008**
	2	**1.15 (1.08, 1.22)**	** < 0.001**	1.03 (0.98, 1.08)	0.242	1.06 (0.94, 1.20)	0.345	1.07 (0.99, 1.15)	0.107
Fat mass	1	**1.10 (1.04, 1.17)**	**0.001**	1.03 (0.99, 1.08)	0.143	**1.14 (1.03, 1.27)**	**0.012**	1.04 (0.96, 1.13)	0.357
	2	**1.09 (1.03, 1.16)**	**0.004**	1.02 (0.97, 1.08)	0.341	**1.16 (1.03, 1.31)**	**0.012**	1.01 (0.93, 1.10)	0.764
Hip BMD	1	**1.12 (1.06, 1.18)**	** < 0.001**	**1.05 (1.01, 1.10)**	**0.027**	**1.13 (1.02, 1.24)**	**0.016**	1.00 (0.93, 1.07)	0.960
	2	**1.09 (1.03, 1.16)**	**0.002**	**1.07 (1.02, 1.12)**	**0.007**	**1.12 (1.01, 1.24)**	**0.035**	0.98 (0.92, 1.06)	0.679

Table shows risk of adverse outcome per SD greater rate of decline in predictor

Conditional changes (independent of baseline) were derived from Years 1 to 4 (Years 2 to 4 for gait speed and Years 1 to 3 for hip BMD). An indicator variable for the corresponding outcomes occurring before the survival analysis follow-up was used as the stratification variable in Cox models for low trauma fracture and hospital admission; models for recurrent falls were adjusted for previous recurrent falls. Model 1: Adjusted for the four-level sex-ethnicity variable and age. Model 2: Additionally adjusted for height, weight-for-height residual, smoking status (ever vs never), alcohol consumption, healthy eating index, physical activity, educational attainment, home ownership, cognitive function, and number of comorbidities

*HR* hazard ratio (odds ratios from a generalized estimating equations model are presented for recurrent falls), *SD* standard deviation, *ALM* appendicular lean mass, *BMD* bone mineral density

Significant associations (*p* < 0.05) are highlighted in bold

Lower baseline values and greater declines in all parameters (excluding the fully-adjusted association for hip BMD level) were associated with increased rates of mortality. For example, fully-adjusted hazard ratios (95% CI) for mortality per SD lower level of gait speed, grip strength and ALM were 1.27 (1.19, 1.36), 1.14 (1.07, 1.21), and 1.17 (1.08, 1.26), respectively; corresponding estimates per SD greater decline in these parameters were 1.19 (1.12, 1.26), 1.09 (1.03, 1.16), and 1.15 (1.08, 1.22). Risk factors of hospital admission included lower levels and greater declines in gait speed and grip strength, and greater declines in hip BMD. Lower levels and greater declines in fat mass and hip BMD were related to increased risk of low trauma fracture. Lower levels of gait speed, higher levels of fat mass, and both lower levels and greater declines in grip strength were associated with greater risk of recurrent falls. All these associations were robust after adjustment for sex, ethnicity, and age and in fully-adjusted analysis.

### Relative Contribution of Baseline Values and Change in Sarcopenia Components to Rates of Mortality

Of the variation in mortality explained by a model including grip strength level and change as predictors, grip strength level explained around 50% of this variation; corresponding figures for gait speed and ALM level were 70% and 20%, respectively.

### Sensitivity Analyses

Determinants of greater numbers of hospital admissions, both after adjustment for sex, ethnicity and age and in fully-adjusted analysis, were lower gait speed, grip strength, and hip BMD and greater declines in gait speed (data not shown). Descriptive statistics for competing risk analyses are presented in eTable 1 (Online Resource). Some associations for hospital admission and low trauma fracture were attenuated when investigated using competing risk models (eTable 2 in Online Resource); however, some key findings such as relationships between lower gait speed and grip strength in relation to increased risk of hospital admission and regarding lower fat mass and hip BMD in relation to greater risk of low trauma fracture were robust in these sensitivity analyses. Baseline levels and changes in parameters were most strongly associated with underlying causes of mortality that were not cardiovascular- or cancer-related, followed by cardiovascular-related mortality and then cancer-related mortality (eTables 3, 4 and 5 in Online Resource). Results for sensitivity analyses relating to ALM (use of ALM residuals and adjusting changes in ALM for changes in body weight) were similar to those from the main analyses (data not shown).

## Discussion

Among participants of the Health ABC Study, we have examined baseline levels and changes in sarcopenia components in relation to rates of mortality, hospital admission, low trauma fracture, and recurrent falls over a subsequent follow-up period ranging from 10 to 14 years. Lower gait speed and grip strength were associated with increased rates of mortality, hospital admission, and recurrent falls; declines in these parameters were related to increased rates of mortality and hospital admission. In contrast, robust relationships regarding levels and changes in ALM were only observed in relation to mortality.

The magnitude of associations differed for the baseline level and change exposures. For baseline levels and changes in gait speed and grip strength in relation to mortality and hospital admission, effect sizes for baseline levels were higher than the corresponding effect sizes for rates of change (Fig. 1). Regarding mortality, hazard ratios were greater for baseline levels of gait speed compared to grip strength (fully-adjusted hazard ratios with 95% CI 1.27 [1.19, 1.36] vs 1.14 [1.07, 1.21]) whereas these were similar for hospital admission (gait speed: 1.14 [1.08, 1.21], grip strength 1.13 [1.07, 1.19]). The relative contribution of grip strength level and change for explaining variation in rates of mortality was similar; the relative contribution was higher for gait speed level compared to gait speed change and lower for ALM level compared to ALM change.

Previous studies have examined levels and changes in gait speed, grip strength and ALM in relation to risk of mortality. The relationships between lower levels and greater declines in grip strength and increased mortality risk are widely established [4, 32–35]. Furthermore, previous literature has established low gait speed [5, 36, 37] and greater declines in gait speed [6, 38] as risk factors for mortality. The relationship between lower ALM and increased mortality risk was reported in the Cardiovascular Health Study [39], and greater declines in ALM in relation to higher risk of mortality were found in the MINOS study, comprising older men [40], and in a previous Health ABC analysis over a shorter follow-up duration [31]; the associations reported in the previous Health ABC analysis were not robust to adjustment for changes in weight.

Levels and changes in these parameters in relation to risk of hospital admission, fractures, and falls have also been explored previously. Lower grip strength was related to increased risk of osteoporotic fracture in a meta-analysis of the MrOS Study [41]; was related to risk of hospital admission in a previous analysis of the Health ABC Study over a shorter follow-up duration of 5 years [23]; and was predictive of emergency and long-stay hospital admission among men and women from the Hertfordshire Cohort Study [42]. Similar to our findings, upper extremity weakness was associated with increased risk of falls and recurrent falls in a systematic review and meta-analysis [43], and rate of grip strength decline was related to subsequent falls in the Women's Health and Aging Study (WHAS) II [44].

Similarly to our findings, slower gait speed was related to greater risk of hospital admission in a previous analysis of the Health ABC Study over a shorter follow-up [23] and future falls in the Einstein Aging Study [45]. In contrast to our findings, slower gait speed was a risk factor for osteoporotic fracture among men in MrOS [41], and gait speed decline was related to risk of incident falls and hip fracture in the MOBILIZE Boston Study [46] and in the Study of Osteoporotic Fractures (SOF) [47], respectively.

Robust relationships between ALM levels and outcomes other than mortality were not found in our study. This is in agreement with a previous analysis of the Health ABC Study where lean mass was not related to risk of hospitalization over a shorter follow-up [23]. Findings which differ from our study include associations between lower ALM index and greater risk of fragility fracture among women from the Os des Femmes de Lyon (OFELY) study [48] and major osteoporotic fracture among men in MrOS [41]; lower ALM was only related to higher risk of low trauma fracture after adjustment for potential confounders in our study.

There are several potential mechanisms which may relate impairment in sarcopenia components to increased risk of mortality. Deficits in components of sarcopenia are correlated with low socio-economic position, poor health behaviors, and increased comorbidity [28] which are established risk factors for mortality. However, associations in this study between muscle mass, strength, and function and mortality remain after adjustment for these factors, suggesting that they do not fully explain the associations observed. Another possibility is that physiological processes such as increased oxidative stress, inflammation, and endocrine dysfunction are contributing to age-related declines in sarcopenia components as well as increased mortality risk [36]. As well as sufficient strength, walking speed also requires motor control and involves multiple anatomical systems; low gait speed may therefore reflect impairment in these systems, leading to greater risk of mortality [5].

Overall our findings are broadly in agreement with the published literature; possible reasons for discrepancies may be due to differences relating to outcomes (such as use of recurrent falls as opposed to incident falls), adjustments, methods used to derive change measures, and follow-up times. However, a key strength of this study is the inclusion of a wide range of musculoskeletal parameters and adverse health outcomes in a single, well-characterized cohort. This enables a comparison of the magnitude of associations between baseline levels and rates of decline in these parameters in relation to clinical outcomes. In contrast, the comparability of associations from previous literature may be reduced due to differences in age ranges and nationalities of participants between studies. Although there are limitations of deriving changes using data from two time-points [49], strengths of the statistical approach implemented include the use of conditional changes in parameters that have zero correlation with baseline levels; a long survival analysis follow-up time (10–14 years) occurring after measurement of exposures; and sensitivity analyses for hospital admission and low trauma fracture which account for the competing risk of death.

A limitation of this study is that participants had no mobility disability at baseline. This limits the generalizability of the findings to the wider population of community-dwelling older people in this age range. Similarly, the exclusion of grip strength values from participants with severe hand pain or recent surgery may have introduced bias as weak grip strength is an established risk factor for adverse health outcomes. In addition, the analyses were restricted to participants with both baseline and change measures for at least one of the parameters of interest, meaning that participants who died before Year 4 (Year 3 for hip BMD) were excluded from the analysis, resulting in attrition bias. These limitations may have led to an underestimation in the magnitude of the reported associations. However, our analyses were internal to this sample; substantial bias should only have occurred if the associations examined differed markedly between Health ABC participants who were and were not included in the analysis; this seems unlikely. Finally, as in all observational studies, there is the possibility that the associations observed may be explained by residual confounding.

Our findings have important clinical implications. The strong associations between slower gait speed and weaker grip strength and multiple adverse health outcomes concur with the position of the Sarcopenia Definitions and Outcomes Consortium (SDOC) that these components, rather than lean mass, should be included in a definition of sarcopenia due to their greater capacity to predict clinically relevant outcomes. For the same reason, the revised definition of the European Working Group on Sarcopenia in Older People (EWGSOP2) regards low muscle strength, rather than lean mass, as the primary component of sarcopenia. Lower baseline values and greater declines in gait speed and grip strength were related to increased rates of mortality and hospital admission in our study. Therefore, interventions aimed at maximizing levels attained in earlier adult life as well as reducing rates of decline from midlife onwards may reduce the burden of disease in this age group as well as improving musculoskeletal health in older age. There is evidence that some musculoskeletal parameters, such as grip strength, have differential determinants for level and change [50] so a focus on both sets of determinants is important for intervention strategies.

## Conclusion

In this study, both lower levels and greater declines in gait speed and grip strength were associated with mortality and hospital admission, with larger effect sizes for levels than rates of change. This suggests that the combined use of absolute levels of parameters as well as rates of change over time could be used to improve the identification of individuals most at risk of adverse outcomes and who are likely to benefit most from interventions.

## Electronic supplementary material

Below is the link to the electronic supplementary material.

Electronic supplementary material 1 (DOCX 27 kb)
