# Supplementary material for: Relationships Between Level and Change in Sarcopenia and Other Body Composition Components and Adverse Health Outcomes: Findings from the Health, Aging, and Body Composition Study
Source: Calcif Tissue Int. 2020 Nov 15;108(3):302–13. doi: 10.1007/s00223-020-00775-3 (PMC7881954; doi:10.1007/s00223-020-00775-3)
Supplement: Supplementary file 1 — Electronic supplementary material 1 (DOCX 27 kb) [file 223_2020_775_MOESM1_ESM.docx]

**Online Resource**

**Article title:** Relationships between level and change in sarcopenia and other body composition components and adverse health outcomes: findings from the Health, Aging and Body Composition Study

**Journal:** Calcified Tissue International and Musculoskeletal Research

**Authors**: LD Westbury, HE Syddall, NR Fuggle, EM Dennison, NC Harvey, JA Cauley, EJ Shiroma, RA Fielding, AB Newman, C Cooper

**Affiliations and e-mail address for corresponding author (Cyrus Cooper)**:

MRC Lifecourse Epidemiology Unit, University of Southampton, Southampton, UK

NIHR Southampton Biomedical Research Centre, University of Southampton and University Hospital Southampton NHS Foundation Trust, Southampton, UK

NIHR Oxford Biomedical Research Centre, University of Oxford, Oxford, UK

cc@mrc.soton.ac.uk

| **eTable 1: Descriptive statistics for competing risk analysis** | | | | | | |
| --- | --- | --- | --- | --- | --- | --- |
|  |  | | |  | | |
| **Characteristic [N(%)]** | **Men** | | | **Women** | | |
|  | **White**  **(n=789)** | **Black**  **(n=398)** | **All**  **(n=1187)** | **White**  **(n=732)** | **Black**  **(n=561)** | **All**  **(n=1293)** |
| *Low trauma fracture* |  |  |  |  |  |  |
| No fracture or death | 325 (42.0%) | 147 (37.0%) | 472 (40.3%)^†^ | 290 (41.4%) | 252 (46.4%) | 542 (43.6%)^†^ |
| Death and no fracture* | 342 (44.2%) | 227 (57.2%) | 569 (48.6%)^†^ | 202 (28.8%) | 228 (42.0%) | 430 (34.6%)^†^ |
| Fracture | 106 (13.7%) | 23 (5.8%) | 129 (11.0%)^†^ | 209 (29.8%) | 63 (11.6%) | 272 (21.9%)^†^ |
|  |  |  |  |  |  |  |
| *Hospital admission* |  |  |  |  |  |  |
| No admission or death | 60 (7.6%) | 29 (7.3%) | 89 (7.5%) | 94 (12.8%) | 69 (12.3%) | 163 (12.6%) |
| Death and no admission* | 34 (4.3%) | 24 (6.0%) | 58 (4.9%) | 21 (2.9%) | 16 (2.9%) | 37 (2.9%) |
| Admission | 695 (88.1%) | 345 (86.7%) | 1040 (87.6%) | 617 (84.3%) | 476 (84.8%) | 1093 (84.5%) |
| ^†^Statistically significant racial differences within sex (p<0.05); differences between sexes were significant (p<0.05) for all characteristics | | | | | | |
| *Represent competing events as death prevents the failure event of interest from occurring | | | | | | |
| Statistics presented for follow-up time starting at Year 4 among 2480 individuals with level and change measures for at least one of the following parameters: gait speed, grip strength, ALM and fat mass; these statistics differ for hip BMD as the exposure where follow-up time started at Year 3 | | | | | | |

| **eTable 2: Risk of adverse outcomes per SD lower baseline level and per SD greater decline in each predictor (estimated using competing risk models with death as the competing event)** | | | | | | | | | | |
| --- | --- | --- | --- | --- | --- | --- | --- | --- | --- | --- |
|  | | | | | | | | | | |
| **Predictor** | **Model** | **Lower baseline level** | | | | **Greater decline** | | | | |
|  |  | **Hospital admission** | | **Low trauma fracture** | | **Hospital admission** | | **Low trauma fracture** | | |
|  |  | **HR (95% CI)** | **P-value** | **HR (95% CI)** | **P-value** | **HR (95% CI)** | **P-value** | **HR (95% CI)** | **P-value** | |
| Gait speed | 1 | **1.17 (1.11,1.23)** | **<0.001** | 1.05 (0.94,1.17) | 0.423 | **1.09 (1.04,1.15)** | **<0.001** | 0.99 (0.89,1.11) | 0.897 | |
|  | 2 | **1.14 (1.07,1.21)** | **<0.001** | 1.06 (0.94,1.19) | 0.366 | **1.08 (1.03,1.14)** | **0.003** | 0.98 (0.87,1.10) | 0.729 | |
|  |  |  |  |  |  |  |  |  |  | |
| Grip strength | 1 | **1.09 (1.04,1.14)** | **<0.001** | **1.14 (1.02,1.26)** | **0.021** | 1.04 (1.00,1.09) | 0.076 | 1.05 (0.94,1.17) | 0.409 | |
|  | 2 | **1.14 (1.08,1.20)** | **<0.001** | 1.09 (0.96,1.24) | 0.182 | 1.04 (0.99,1.09) | 0.103 | 1.04 (0.92,1.17) | 0.512 | |
|  |  |  |  |  |  |  |  |  |  | |
| ALM | 1 | 1.00 (0.95,1.04) | 0.859 | 1.09 (0.96,1.23) | 0.194 | 1.04 (0.99,1.09) | 0.103 | 1.02 (0.91,1.15) | 0.700 | |
|  | 2 | 1.03 (0.97,1.09) | 0.371 | 1.14 (0.96,1.34) | 0.128 | 1.03 (0.98,1.08) | 0.293 | 1.02 (0.91,1.16) | 0.701 | |
|  |  |  |  |  |  |  |  |  |  | |
| Fat mass | 1 | 0.97 (0.93,1.02) | 0.232 | **1.16 (1.03,1.30)** | **0.011** | 1.03 (0.98,1.08) | 0.197 | **1.10 (1.00,1.22)** | **0.049** | |
|  | 2 | 0.99 (0.94,1.03) | 0.557 | **1.17 (1.03,1.33)** | **0.017** | 1.02 (0.97,1.08) | 0.398 | **1.13 (1.01,1.26)** | **0.033** | |
|  |  |  |  |  |  |  |  |  |  | |
| Hip BMD | 1 | 1.02 (0.98,1.07) | 0.388 | **1.73 (1.54,1.93)** | **<0.001** | 1.04 (0.99,1.08) | 0.138 | 1.09 (0.99,1.20) | 0.088 | |
|  | 2 | 1.05 (0.99,1.11) | 0.088 | **1.96 (1.69,2.27)** | **<0.001** | 1.05 (0.99,1.10) | 0.082 | 1.09 (0.98,1.21) | 0.120 | |
| HR: Hazard ratio; SD: Standard deviation; ALM: Appendicular lean mass; BMD: Bone mineral density | | | | | | | | | | |
| Baseline levels ascertained at Year 2 for gait speed and at Year 1 for remaining predictors; conditional changes (independent of baseline) were derived from Years 1 to 4 (Years 2 to 4 for gait speed and Years 1 to 3 for hip BMD) | | | | | | | | | | |
| An indicator variable for the corresponding outcomes occurring before the survival analysis follow-up was used as an adjustment in models | | | | | | | | | | |
| Model 1: Adjusted for the four-level sex-ethnicity variable and age | | | | | | | | | | |
| Model 2: Additionally adjusted for height, weight-for height residual (not used in models for level relating to ALM and fat mass), smoking status (ever vs never), alcohol consumption, healthy eating index, physical activity, educational attainment, home ownership, cognitive function and number of comorbidities | | | | | | | | | | |
| Significant associations (p<0.05) are highlighted in bold | | | | | | | | | | |

| **eTable 3: Descriptive statistics for underlying cause of death among participants who died during follow-up** | | | | | | |
| --- | --- | --- | --- | --- | --- | --- |
|  |  | | |  | | |
| **Underlying cause of death [N(%)]** | **Men** | | | **Women** | | |
|  | **White**  **(n=510)** | **Black**  **(n=282)** | **All**  **(n=792)** | **White**  **(n=387)** | **Black**  **(n=328)** | **All**  **(n=715)** |
|  |  |  |  |  |  |  |
| Cardiovascular-related | 178 (35.0%) | 100 (35.5%) | 278 (35.1%) | 135 (34.9%) | 119 (36.3%) | 254 (35.5%) |
| Cancer-related | 116 (22.8%) | 78 (27.7%) | 194 (24.5%) | 76 (19.6%) | 74 (22.6%) | 150 (21.0%) |
| Other | 215 (42.2%) | 104 (36.9%) | 319 (40.3%) | 176 (45.5%) | 135 (41.2%) | 311 (43.5%) |
|  |  |  |  |  |  |  |
| No statistically significant ethnic differences within sex or differences between sexes were observed (p>0.05 for all associations) | | | | | | |
| One white man who died had missing information regarding cause of death | | | | | | |
| Statistics presented for follow-up time starting at Year 4 among participants with level and change measures for at least one of the following parameters: gait speed, grip strength, ALM and fat mass; these statistics differ for hip BMD as the exposure where follow-up time started at Year 3 | | | | | | |

| **eTable 4: Muscle function, body composition and underlying cause of death: impact of baseline indices**  ***Table shows risk of adverse outcome per SD lower baseline level of predictor*** | | | | | | | | | |  |  |
| --- | --- | --- | --- | --- | --- | --- | --- | --- | --- | --- | --- |
|  | | | | | | | | | |  |  |
| **Predictor** | **Model** | **All-cause** | | **Cardiovascular-related** | | **Cancer-related** | | **Other** | |  |  |
|  |  | **HR (95% CI)** | **P-value** | **HR (95% CI)** | **P-value** | **HR (95% CI)** | **P-value** | **HR (95% CI)** | **P-value** |  |  |
| Gait speed | 1 | **1.31 (1.24,1.39)** | **<0.001** | **1.37 (1.24,1.51)** | **<0.001** | 1.10 (0.98,1.25) | 0.116 | **1.39 (1.27,1.53)** | **<0.001** |  |  |
|  | 2 | **1.27 (1.19,1.36)** | **<0.001** | **1.36 (1.22,1.52)** | **<0.001** | 1.07 (0.93,1.23) | 0.350 | **1.32 (1.19,1.47)** | **<0.001** |  |  |
|  |  |  |  |  |  |  |  |  |  |  |  |
| Grip strength | 1 | **1.13 (1.07,1.20)** | **<0.001** | **1.15 (1.05,1.26)** | **0.003** | 0.95 (0.85,1.06) | 0.335 | **1.24 (1.14,1.35)** | **<0.001** |  |  |
|  | 2 | **1.14 (1.07,1.21)** | **<0.001** | **1.15 (1.03,1.28)** | **0.011** | 0.98 (0.86,1.12) | 0.775 | **1.23 (1.11,1.36)** | **<0.001** |  |  |
|  |  |  |  |  |  |  |  |  |  |  |  |
| ALM | 1 | **1.07 (1.01,1.14)** | **0.018** | 1.05 (0.96,1.16) | 0.299 | 0.96 (0.86,1.08) | 0.535 | **1.16 (1.06,1.27)** | **0.001** |  |  |
|  | 2 | **1.17 (1.08,1.26)** | **<0.001** | **1.17 (1.03,1.33)** | **0.013** | 1.04 (0.89,1.20) | 0.630 | **1.25 (1.11,1.40)** | **<0.001** |  |  |
|  |  |  |  |  |  |  |  |  |  |  |  |
| Fat mass | 1 | **1.06 (1.00,1.12)** | **0.036** | 1.03 (0.94,1.13) | 0.486 | 0.94 (0.84,1.05) | 0.256 | **1.17 (1.07,1.28)** | **<0.001** |  |  |
|  | 2 | **1.12 (1.06,1.20)** | **<0.001** | **1.12 (1.00,1.24)** | **0.041** | 0.98 (0.87,1.10) | 0.705 | **1.24 (1.12,1.37)** | **<0.001** |  |  |
|  |  |  |  |  |  |  |  |  |  |  |  |
| Hip BMD | 1 | **1.09 (1.03,1.15)** | **0.002** | 1.05 (0.96,1.15) | 0.258 | 0.97 (0.87,1.08) | 0.596 | **1.20 (1.10,1.31)** | **<0.001** |  |  |
|  | 2 | 1.06 (0.99,1.14) | 0.070 | 1.07 (0.95,1.19) | 0.272 | 0.99 (0.86,1.13) | 0.858 | 1.11 (0.99,1.23) | 0.066 |  |  |
| HR: Hazard ratio; SD: Standard deviation | | | | | | | | | | | |
| ALM: Appendicular lean mass; BMD: Bone mineral density | | | | | | | | | | | |
| Underlying causes of death that were not cardiovascular-related or cancer-related were classed as ‘Other’ | | | | | | | | | | | |
| Baseline levels ascertained at Year 2 for gait speed and at Year 1 for remaining predictors | | | | | | | | | | | |
| Model 1: Adjusted for the four-level sex-ethnicity variable and age | | | | | | | | | | | |
| Model 2: Additionally adjusted for height, weight-for-height residual (not used in models for ALM and fat mass), smoking status (ever vs never), alcohol consumption, healthy eating index, physical activity, educational attainment, home ownership, cognitive function and number of comorbidities | | | | | | | | | | | |
| Significant associations (p<0.05) are highlighted in bold | | | | | | | | | | | |

| **eTable 5: Muscle function, body composition and underlying cause of death: impact of rates of loss**  ***Table shows risk of adverse outcome per SD greater rate of decline in predictor*** | | | | | | | | | |  |  |
| --- | --- | --- | --- | --- | --- | --- | --- | --- | --- | --- | --- |
|  | | | | | | | | | |  |  |
| **Predictor** | **Model** | **All-cause** | | **Cardiovascular-related** | | **Cancer-related** | | **Other** | |  |  |
|  |  | **HR (95% CI)** | **P-value** | **HR (95% CI)** | **P-value** | **HR (95% CI)** | **P-value** | **HR (95% CI)** | **P-value** |  |  |
| Gait speed | 1 | **1.21 (1.14,1.28)** | **<0.001** | **1.22 (1.12,1.34)** | **<0.001** | **1.16 (1.03,1.30)** | **0.012** | **1.22 (1.12,1.33)** | **<0.001** |  |  |
|  | 2 | **1.19 (1.12,1.26)** | **<0.001** | **1.20 (1.09,1.32)** | **<0.001** | 1.12 (0.99,1.26) | 0.068 | **1.22 (1.12,1.34)** | **<0.001** |  |  |
|  |  |  |  |  |  |  |  |  |  |  |  |
| Grip strength | 1 | **1.14 (1.08,1.20)** | **<0.001** | **1.22 (1.12,1.33)** | **<0.001** | 0.96 (0.86,1.07) | 0.431 | **1.18 (1.09,1.28)** | **<0.001** |  |  |
|  | 2 | **1.09 (1.03,1.16)** | **0.002** | **1.17 (1.07,1.29)** | **0.001** | 0.92 (0.82,1.04) | 0.173 | **1.14 (1.04,1.24)** | **0.004** |  |  |
|  |  |  |  |  |  |  |  |  |  |  |  |
| ALM | 1 | **1.17 (1.11,1.24)** | **<0.001** | 1.07 (0.97,1.17) | 0.170 | **1.19 (1.06,1.33)** | **0.003** | **1.26 (1.15,1.37)** | **<0.001** |  |  |
|  | 2 | **1.15 (1.08,1.22)** | **<0.001** | 1.03 (0.93,1.14) | 0.536 | **1.19 (1.05,1.34)** | **0.006** | **1.23 (1.12,1.35)** | **<0.001** |  |  |
|  |  |  |  |  |  |  |  |  |  |  |  |
| Fat mass | 1 | **1.10 (1.04,1.17)** | **0.001** | 1.06 (0.97,1.17) | 0.190 | 1.02 (0.91,1.14) | 0.770 | **1.20 (1.10,1.30)** | **<0.001** |  |  |
|  | 2 | **1.09 (1.03,1.16)** | **0.004** | 1.06 (0.96,1.18) | 0.246 | 0.99 (0.87,1.12) | 0.845 | **1.20 (1.09,1.32)** | **<0.001** |  |  |
|  |  |  |  |  |  |  |  |  |  |  |  |
| Hip BMD | 1 | **1.12 (1.06,1.18)** | **<0.001** | **1.11 (1.02,1.21)** | **0.012** | 1.07 (0.96,1.19) | 0.205 | **1.15 (1.06,1.25)** | **0.001** |  |  |
|  | 2 | **1.09 (1.03,1.16)** | **0.002** | **1.10 (1.00,1.20)** | **0.048** | 1.04 (0.93,1.16) | 0.509 | **1.12 (1.03,1.23)** | **0.009** |  |  |
| HR: Hazard ratio; SD: Standard deviation | | | | | | | | | | | |
| ALM: Appendicular lean mass; BMD: Bone mineral density | | | | | | | | | | | |
| Underlying causes of death that were not cardiovascular-related or cancer-related were classed as ‘Other’ | | | | | | | | | | | |
| Conditional changes (independent of baseline) were derived from Years 1 to 4 (Years 2 to 4 for gait speed and Years 1 to 3 for hip BMD) | | | | | | | | | | | |
| Model 1: Adjusted for the four-level sex-ethnicity variable and age | | | | | | | | | | | |
| Model 2: Additionally adjusted for height, weight-for-height residual, smoking status (ever vs never), alcohol consumption, healthy eating index, physical activity, educational attainment, home ownership, cognitive function and number of comorbidities | | | | | | | | | | | |
| Significant associations (p<0.05) are highlighted in bold | | | | | | | | | | | |
